# Editorial: NK/T-Cell Lymphoma: Biology, Prognostics, Prediction, and Treatment

**DOI:** 10.3389/fonc.2022.942299

**Published:** 2022-06-17

**Authors:** Jean El Cheikh, Khodr Terro, Layal Sharrouf, Anjali Mishra, Marcos De Lima

**Affiliations:** ^1^ Division of Hematology/Oncology, Department of Internal Medicine, American University of Beirut Medical Center, Beirut, Lebanon; ^2^ Bone Marrow Transplantation Program, Department of Internal Medicine, American University of Beirut Medical Center, Beirut, Lebanon; ^3^ Division of Hematologic Malignancies and Hematopoietic Stem Cell Transplantation, Department of Medical Oncology, Sidney Kimmel Cancer Center, Philadephia, PA, United States; ^4^ Department of Medicine, Division of Hematology, The Ohio State University, Columbus, OH, United States

**Keywords:** NK/T-cell lymphoma: biology, prognostics, treatment, diagnosis, prediction

The Research Topic at hand incorporates a sum of contributions, from our colleagues in South Korea and China mainly, which shed light on the biology, prognostication, prediction, risk assessment and treatment of a rare subtype of Epstein-Barr virus (EBV)-related non-Hodgkin lymphomas, NK/T-cell lymphoma (NKTL).

Although the overall incidence of NKTL is low, it has a higher incidence in certain parts of the world, especially in Asia and to a lesser extent, in Latin America. It mainly affects the midline area unfolding as a necrotic granulomatous and potentially extremely disfiguring lesion. The most common subtype of NKTL is nasal which appears in the nasal cavity including the nasopharynx, oropharynx, parts of the aero digestive tract and Waldeyer’s ring. The other subtypes are collectively referred to as non-nasal NK/T cell lymphoma which involves sites like the skin, testis, gastrointestinal tract, salivary glands and muscle. NKTL frequently expresses multidrug resistance-associated P-glycoprotein, which mediates exporting many antitumor agents outside tumor cells. (Terro et al.) 

The prognosis of localized NKTL is significantly better than when the disease is relapsed and refractory (RR). (Gao et al.) suggests a new model of Prognostic Index of Natural Killer Lymphoma (PINK) named PINK+PD-L1 by including PD-L1 expression as an additional risk factor. PINK+PD-L1 model effectively identified RR patients from a population of NKTL patients, and a 25% lower expression of PD-L1 was shown to be independently associated with RR subtypes of NKTL patients (P=0.0019). In addition, higher expression of mutations like JAK-STAT, PI3K-AKT, and NF-kappa B was found in RR patients compared to successfully treated patients. Early identification of RR disease may hopefully lead to early adoption of improved therapies for this subset of patients thus ultimately improving their prognosis.

The presence of EBV DNA is a well-established prognostic factor in NKTL patients. However, (Ha et al.) took it a step further by trying to identify which blood compartment in specific is better at predicting the clinical outcomes of NKTL. EBV DNA was identified in whole blood (WB) and in plasma at both times before and after treatment. Both pre and post-treatment positive plasma EBV were observed to be significantly and independently associated with poor progression free survival (PFS) and overall survival (OS) rates. On the other hand, there was no significant association observed between WB EBV and survival outcomes in NKTL patients, suggesting that plasma EBV DNA has a superior prognostic value over WB EBV DNA in NKTL patients.

Patients with hematologic malignancies including NKTL are at a higher risk of developing secondary hemophagocytic lymphohistiocytosis (HLH) also known as NK/T cell lymphoma-associated hemophagocytic syndrome (NK/T-LAHS). NK/T-LAHS is a pathologic activation of the immune system with a rapidly fatal course which further worsens the prognosis of NKTL. (Li et al.) find it essential to develop a way to predict NK/T-LAHS in NKTL patients to implement a risk adapted treatment thus improving patients’ prognosis. In their research article they demonstrated that poor Eastern Cooperative Oncology Group (ECOG) performance status, B symptoms and bone marrow invasion were collectively significantly associated with the occurrence of NK/T-LAHS. The authors developed their first risk model called risk index for NKTL (RINK). This was followed by a second risk model called risk index for NKTL-Epstein-Barr virus (RINK-E) which included high viral EBV-DNA copies (≥4,450 copies/ml) as an additional risk factor to RINK. Both nomograms RINK and RINK-E were validated in a large cohort of NKTL patients and were highly accurate at predicting NK/T-LAHS.

This Research Topic also includes a retrospective study by (Liu et al.), which tackled the prognosis, survival and treatment outcomes in a cohort of 336 advanced stage NKTL patients. This study has contributed effectively to the limited pool of data existing on such a rare subtype of NKTL patients. Patients treated with asparaginase containing chemotherapy showed better overall response rate (65.2% vs. 47.8%, *P* = 0.006) and better complete remission rates (45.5% vs. 23.9%, *P* < 0.001) than patients treated without Asparaginase-containing regimens. Moreover, 5-year PFS and OS rates were improved by asparaginase containing regimens (17.1% versus 34.2%, *P* < 0.001) and (27.8% versus 45.3% *P* < 0.001) respectively. Addition of gemcitabine to asparaginase-containing regimens showed even further improvement in OS.

A very promising new chemotherapy regimen for naïve NKTL patients was studied by (Wang et al.) in a phase II clinical trial. The authors reported on 36 newly diagnosed NKTL patients who were treated with a combination regimen ‘GAD-M’, consisting of peg-asparaginase, gemcitabine, and methotrexate. Administering GAD-M regimen along with radiotherapy lead to compelling 5-year PFS and OS rates of 68.3% and 77.8% respectively. Hence, GAD-M could be considered a treatment option for such patients

A new salvage regimen is proposed in a case report of an elderly female patient with treatment resistant NKTL. (Xia et al.) treated a patient with EBV-associated NKTL who failed to respond to cyclophosphamide, doxorubicin, vindesine, and prednisone regimen. However, she achieved a complete response after three cycles of anti-PD-1 antibody (tislelizumab) combined with gemcitabine and oxaliplatin (GemOx) regimen.

We hope the reader will enjoy this Research Topic, and find it to be a useful reference for the multiple ongoing advancements in prognostication, treatment, risk assessment and management of NKTL patients.

## Author Contributions

JC is the editor of this topic, invited the authors and reviewers for all the submitted manuscript. KT and LS contributed for the editorial, AM and ML are the co-editor of this topic. All named authors meet the International Committee of Medical Journal Editors (ICMJE) criteria for authorship for this manuscript, take responsibility for the integrity of the work as a whole, and have given final approval to the version to be published.

## Conflict of Interest

The authors declare that the research was conducted in the absence of any commercial or financial relationships that could be construed as a potential conflict of interest.

## Publisher’s Note

All claims expressed in this article are solely those of the authors and do not necessarily represent those of their affiliated organizations, or those of the publisher, the editors and the reviewers. Any product that may be evaluated in this article, or claim that may be made by its manufacturer, is not guaranteed or endorsed by the publisher.

